# Atopic Multimorbidity in Adults With a Focus on Sensitization Patterns and T Cell Activation

**DOI:** 10.1002/clt2.70129

**Published:** 2025-11-28

**Authors:** Ariane Bialas, Marie Rabe, Niklas Artz, Andreas Boldt, Jan C. Simon, Regina Treudler, Benjamin Klein

**Affiliations:** ^1^ Department of Dermatology, Allergology and Venereology Leipzig University Medical Center, University of Leipzig Leipzig Germany; ^2^ Institute of Clinical Immunology, University of Leipzig Leipzig Germany; ^3^ Institute of Allergology, Charité—Universitätsmedizin Berlin, Corporate Member of Freie Universität Berlin and Humboldt‐Universität zu Berlin Berlin Germany

**Keywords:** allergy, atopic dermatitis, atopy, type 2 immunity, urticaria

## Abstract

**Background:**

Atopic diseases—including atopic dermatitis (AD), asthma (AA), and allergic rhinitis (AR)—are driven by Th2 inflammation and often occur together (atopic multimorbidity), along with non‐atopic comorbidities. Chronic spontaneous urticaria (CSU) is an autoimmune mast cell‐driven disease, but its relationship to classic atopic diseases remains unclear. This study investigated the association of CSU with classical atopic diseases as well as sensitization patterns and T cell activation in atopic multimorbidity.

**Methods:**

We conducted a prospective, single‐center study involving 123 participants who completed structured questionnaires regarding physician‐diagnosed AD, AA, AR, and/or CSU, as well as non‐atopic comorbidities and a history of type I sensitizations. AD patients (*n* = 22, with or without AR/AA, but not CSU) and healthy controls (*n* = 20) underwent additional immunophenotyping. Peripheral blood T cell subsets and T cell activation status were measured by flow cytometry and compared across groups.

**Results:**

Individuals with atopic multimorbidity exhibited more frequent type I sensitizations, sleep disorders, and elevated serum IgE levels. CSU differed from classical atopic diseases regarding age of onset and duration and was therefore excluded from immunophenotyping. T cell subsets and activation in AD did not differ by presence of atopic multimorbidity but correlated with disease activity scores.

**Conclusion:**

Our findings highlight the burden associated with atopic multimorbidity, demonstrated by increased serum IgE and sensitization rates in individuals with multiple atopic diseases. Importantly, T cell activation appeared to be more closely related to AD disease activity rather than the presence of classic atopic comorbidities.

## Graphical abstract

Our study looked at atopic dermatitis and atopic comorbidities (rhinitis, asthma) with regards to type 1 allergic sensitizations, serum IgE values and systemic T cell activation. Our results show that IgE values increase with atopic disease burden while T cell activation is rather dependent on disease activity than atopic multimorbidity.

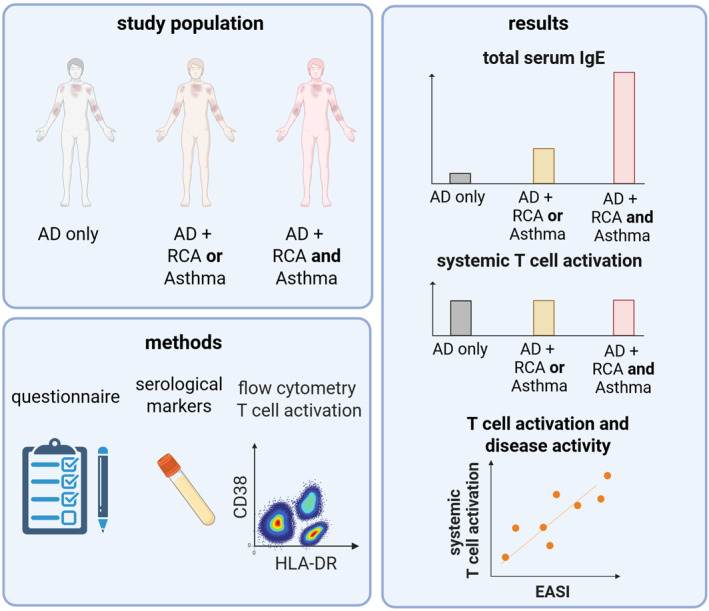

## 1 Introduction

Atopic dermatitis (AD), food allergy, allergic asthma (AA) and allergic rhinits (AR) have been linked to type 2 immunity, driven by Th2/Tc2 cells, Group 2 innate lymphoid cells and mast cells [1]. Cytokines that are involved in the pathogenesis of type 2 immune‐mediated diseases include IL‐4, IL‐5, IL‐13, and IL‐31. These cytokines recruit effector cells to the site of inflammation to further promote Th2 polarization and B cell class switching to immunoglobulin E^1^, leading to allergen sensitization. The close relationship of these diseases is highlighted by the term “atopic march”, which describes the sequential onset of AD, followed by AA and AR in early childhood [2]. But only a subset of patients follows this classic trajectory of the atopic march. The newer term “atopic multimorbidity” defines the condition of having two or more atopic diseases at a time as atopic diseases do not necessarily follow a predetermined trajectory of progression [2, 3]. While activation of T cells has been widely reported in atopic diseases [4, 5, 6], its association to atopic multimorbidity has not been studied.

Chronic spontaneous urticaria (CSU) is a complex mast cell‐driven disease with two main immunological pathways of mast cell activation: One way of activation is driven by cross‐linking of IgE (auto)antibodies (type I) against autoantigens such as thyroid peroxidase. Another way is via IgG (auto)antibodies (type II) against IgE or its receptor FcεRI [7, 8]. However, many more mast cell activation and silencing pathways are known (i.e., MRGPRX2, Siglec 8, chemokine receptors) [8]. Whether CSU is associated with classical atopic diseases remains debatable [9, 10, 11] but type I hypersensitivity with IgE antibodies can be present in both AD and CSU.

Comorbidities in atopic diseases include atopic and non‐atopic disorders [12]. The development of different atopic diseases as well as their overlapping is still a subject of research [3]. Non‐atopic comorbidities have been described in multiple epidemiological studies and include psychological, autoimmune and infectious disorders [13]. We and others previously described increased depression, anxiety, sleep disorders and suicidal ideation among individuals with AD compared to non‐atopic controls [14, 15, 16, 17].

Current data provide limited clarity on the relationship between serological markers of type 2 immunity or systemic T cell activation and atopic disease burden. We conducted a prospective, single center study to test associations of classic atopic diseases with CSU and other comorbidities, assessed allergic type 1 sensitizations, total IgE values as well as T cell activation in patients with one, two or three classic atopic diseases. Our results show that CSU differs from AD, AA and AR regarding age of onset and symptom duration. We show that type I hypersensitivity and IgE values are increased in individuals with atopic multimorbidity. Importantly, we found that T cell activation in AD is rather dependent on disease activity than atopic multimorbidity, suggesting disease‐specific T cell activation signatures.

## 2 Methods

### 2.1 Study Design and Participants for Questionnaire

We conducted a prospective monocentric study conducted at our GA2LEN certified Atopic Dermatitis Center of Reference and Excellence at the Department of Dermatology, Venereology and Allergology at the University Medical Center in Leipzig, Germany. In the period from February 2021 to May 2024, a total of 127 patients with physician‐diagnosed Th2‐associated diseases were included in the allergology consultation hours. The study was approved by the Ethics Committee of the Medical Faculty of the University of Leipzig (552/21‐ek). All participants signed written consent prior to participation. Inclusion criteria were: > 18 years old and presence of a physician‐diagnosed atopic disease based on diagnostic criteria (Hanifin and Rajka for AD [18], Zuberbier for CSU [19]). Four patients were excluded due to lack of responses in the questionnaires. The remaining 123 patients were included in this study. The following diseases were considered: AD (72 mentions), AA (57 mentions), AR (86 mentions) and CSU (29 mentions). Patient‐reported disease activity tools were used for each disease: Atopic Dermatitis Control Tool (ADCT) for AD, Rhinitis Control Assessment Test (RCAT) for AR, Asthma Control Score (ACS) for AA and Urticaria Control Test (UCT) for CSU. We further asked for hypersensitivity reactions against pollen, house dust mite, pet, mold, food, contact allergens and drugs. We also asked for physician‐diagnosed mental and autoimmune comorbidities. Mental comorbidities included depression, anxiety disorder, sleep disorder, attention deficit hyperactivity disorder (ADHD), addiction, anorexia, obsessive compulsive disorder (OCD), personality disorder and others. Regarding autoimmune comorbidities, we asked for autoimmune thyroiditis, vitiligo, alopecia areata, rheumatoid arthritis, diabetes mellitus type I, chronic inflammatory bowel disease, systemic lupus and others. Patients were sub‐categorized based on the number of atopic diseases. Group A (one disease) contained 41 patients, group B (two diseases) 43 patients and group C (three diseases) 39 patients (Table 1). At around 66% overall, significantly more women than men took part in the study, and this female predominance is also reflected in the individual groups.

**TABLE 1:** Patient characteristics for questionnaire‐based data.^a^

# Of atopic diseases	*n*	Age mean (SD)	Body mass index (BMI) mean (SD)	Sex (M = male, F = female)	Counts (%)
One disease	41	39.95 ± 17.02	25.78 ± 4.91	M	10 (24.4)
				F	31 (75.6)
Two diseases	43	37.98 ± 13.4	25.81 ± 6.03	M	14 (32.6)
				F	29 (67.4)
Three diseases	39	38.28 ± 14.24	25.69 ± 7.01	M	17 (43.6)
				F	22 (56.4)

^a^ No significant differences regarding age, BMI or sex distribution.

### 2.2 Peripheral Blood Immunophenotyping

**FIGURE 1:**
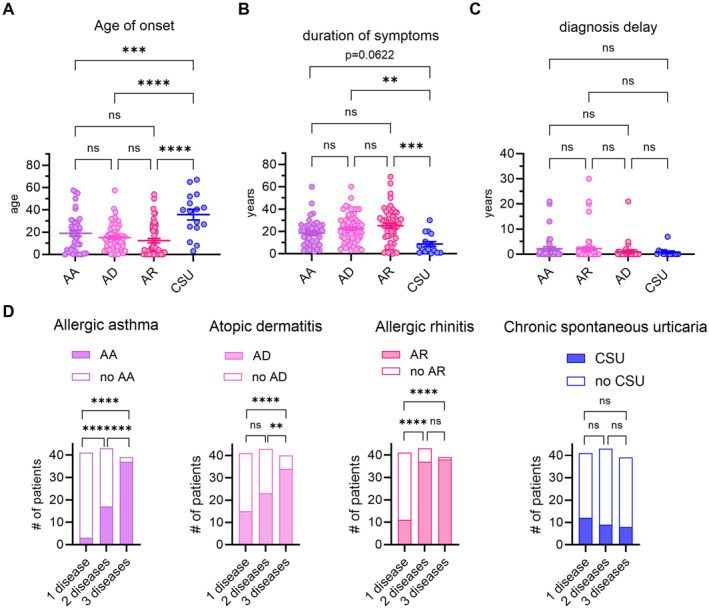
Disease onset in classical atopic diseases differs from CSU. (A–C) Comparison of age of onset (A), duration of symptoms (B) and diagnosis delay (C) in years in physician‐diagnosed allergic asthma (AA), atopic dermatitis (AD), allergic rhinitis (AR) and chronic spontaneous urticaria (CSU). (D) Numbers of patients with AR, AD, AR or CSU in patients with one, two or three atopic diseases. Note that AA, AD and AR increase with higher atopic disease burden while CSU remains a similar number of patients. Mean + SEM. *p* values were calculated using ordinary one‐way analysis of variance (ANOVA) followed by Šidák's multiple comparison test or Fisher's exact test. *p < 0.05, **p < 0.01, ***p < 0.001, and ****p < 0.0001. ns, not significant.

Twenty two patients with AD were chosen for immunophenotyping of peripheral T cells after informed consent. All patients and controls were Caucasian. Adult patients diagnosed according to criteria of Hanifin and Rajka [18] without current systemic treatment and with active skin lesions were recruited through the Department of Dermatology in the comprehensive allergy center at Leipzig University Medical Center, Germany. Patients with CSU were excluded for immunophenotyping due to observed differences compared to AD, AR and AA. Based on the presence of comorbidities, patients were stratified into AD only, AD with one comorbidity (AD+, with AR *or* AA) and AD with two comorbidities (AD++, with AR *and* AA). All AD patients had skin lesions at the time of blood draw that were assessed by Eczema Area and Severity index (EASI). Topical steroids were reportedly used in 68% of the patients, although we only included patients using low‐moderate topical steroids (class VII‐IV). We selected our patient cohort to include a wide variety of disease severity as measured by EASI score for correlation analysis. In addition, we enrolled in 20 age and sex matched healthy controls (HC) defined as absence of skin disease and no current infection and medication. Clinical characteristics of patients and HC can be seen in Table 2. Distribution of age, EASI scores and sensitization patterns in AD only, AD+ and AD++ are shown in Supporting Information S1: Figure 1A,B.

**TABLE 2:** Clinical characteristics of study participants that underwent immunophenotyping.

	AD (*n* = 22)	HC (*n* = 20)
Characteristic	mean (SD) % (counts)	mean (SD) % (counts)
Age (years)	38.6 (± 15.3)	35 (± 10.3)
Female	64% (14/22)	55% (11/20)
Male	36% (8/22)	45% (9/20)
EASI	17.5 (± 12.1)	
EASI range	0.7–42.4	
Atopic dermatitis control tool (ADCT)	15.9 (± 6.5)	
Asthma control score (ACS)	19.7 (± 4.5)	
Rhinitis control assessment test (RCAT)	21.4 (± 4.2)	
Age at AD onset (years)	10.2 (± 12)	
Allergic rhinitis	72% (16/22)	
Allergic asthma	55% (12/22)	
Urticaria	0% (0/22)	
Eosinophilic esophagitis	0% (0/22)	
Sensitization pollen (birch, ragweed)	59% (13/22)	
Sensitization dust mite	68% (15/22)	
Sensitization food	14% (3/22)	
Topical corticosteroid	68% (15/22)	
Systemic corticosteroid	0% (0/22)	
Biological/systemic immunosuppression	0% (0/22)	

Abbreviations: AD, atopic dermatitis, HC, healthy controls.

### 2.3 Flow Cytometry for T Cell Immunophenotyping

For measurement of T cell subsets and T cell activation in our immunophenotyping cohort, whole blood from patients and controls was obtained to assess T‐, B‐ and NK cells, as previously described [20]. T cell activation was measured using CD38 (BV421, clone HIT2, Fa. BD Horizon, 1:40) and HLA‐DR (BV605; clone G46‐6; Fa. BD Horizon, 1:40) stained in CD4^+^ and CD8^+^ T cells. Additionally, T‐Helper subsets were identified by different chemokine receptor surface expressions: Th1: CD3^+^CD4^+^CXCR3^+^CCR6^−^, Th2: CD3^+^CD4^+^CXCR3^−^CCR6^‐^CRTH2^+^ and Th17/22: CD3^+^CD4^+^CXCR3^−^CCR6^+^.

### 2.4 Statistical Analysis

Statistical analysis was performed using GraphPad Prism version 10.1.0. For categorial variables, Fisher's exact *t* test and Chi square test were used. For comparison of quantitative data, we used a two‐tailed Student's test (normally distributed values) or a Mann‒Whitney *U*‐test (not normally distributed values) when comparing two groups. Values with more than two groups were analyzed using 2way ANOVA followed by Šídák's multiple comparisons test. Correlation analysis was performed using Pearson or Spearman correlation coefficient. Significance was determined by a *p* value of < 0.05, and annotated as *p < 0.05, **p < 0.01, ***p < 0.001 and ****p < 0.0001.

## 3 Results

### 3.1 Distinction of Classic Atopic Diseases From Chronic Spontaneous Urticaria

First, we wanted to know how atopic diseases relate to each other in adulthood. After including 123 patients in our study, we were interested whether CSU, which reportedly shares features of atopic diseases, was associated with the manifestation of other classical atopic diseases. No patient of our cohort had all four diseases. We found that the age of onset in CSU was significantly higher than all the other atopic diseases (AA, AD, AR) while we did not identify significant differences within the three classical atopic diseases (Figure 1A). Similarly, duration of symptoms was significantly lower in CSU compared to AD and AR (Figure 1B), and comparison of diagnosis delay did not differ between these four diseases (Figure 1C). This suggests that CSU occurs later in life than other classical atopic diseases.

We then looked at the distribution of each individual atopic diseases when multiple atopic diseases were present. Therefore, we divided our cohort into three groups, dividing patients with one atopic disease (*n* = 41), from patients with two (*n* = 43) and three (*n* = 39) atopic diseases. Here, we saw a gradual increase in classical atopic diseases (AD, AA, AR) when two or three diseases were present whereas CSU did not show this distribution (Figure 1D). This suggests that even though CSU shares some features of type 2 inflammation, it does not follow the classic atopic trajectory (“march”) in our cohort.

### 3.2 Psychological and Autoimmune Comorbidities in Atopic Multimorbidity

**FIGURE 2:**
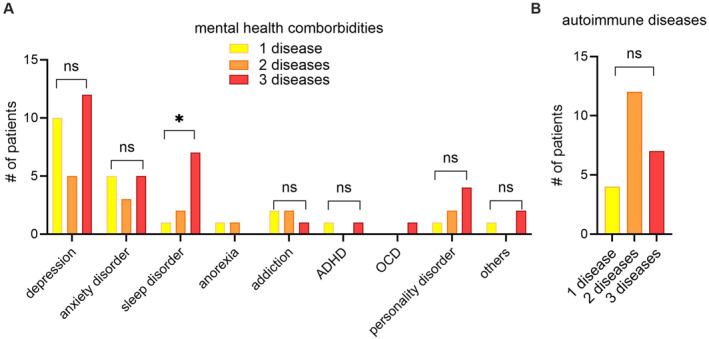
Mental health and autoimmune comorbidity in atopic disease burden. (A–B) Bar plots indicate counts of patients with mental health comorbidities (A) and autoimmune diseases (B). In (B) the following autoimmune diseases were assessed: autoimmune thyroiditis, vitiligo, alopecia areata, rheumatoid arthritis, diabetes mellitus type I, chronic inflammatory bowel disease, systemic lupus and others. These were summarized due to low counts. *p‐*values were calculated using Fisher's exact test comparing counts with nonaffected patients across groups. *p < 0.05, ns, not significant. ADHD, attention‐deficit/hyperactivity disorder; OCD, obsessive‐compulsive disorder.

AD and other atopic disease are known to be associated with psychological comorbidities. We previously described increased rates of depression, anxiety and suicidal ideation in individuals with AD compared to non‐atopic individuals [13, 14]. Here, we wanted to compare psychological comorbidities in patients with atopic multimorbidity. Overall, depression was the most frequent self‐reported psychological comorbidity in our cohort, followed by anxiety (Figure 2A). The number of these diseases did not increase with atopic disease burden. However, sleep disorder was significantly higher in individuals with three atopic diseases compared to one atopic disease, making it the second most frequent psychological comorbidity in patients with three atopic diseases (Figure 2A). We were underpowered to get statistical significance for other psychological disorders. Regarding autoimmune diseases, we observed low self‐reported numbers of individual diseases (autoimmune thyroiditis, vitiligo, alopecia areata, rheumatoid arthritis, diabetes mellitus type I, chronic inflammatory bowel disease, systemic lupus), leading us to summarize autoimmune diseases overall (Figure 2B). We did not observe differences in frequency of autoimmune disorders across one, two or three atopic diseases. Next, we wanted to assess whether atopic multimorbidity exhibits changes in hypersensitivity reactions, laboratory findings or T cell activation.

### 3.3 Hypersensitivity Reactions Increase With Atopic Multimorbidity

**FIGURE 3:**
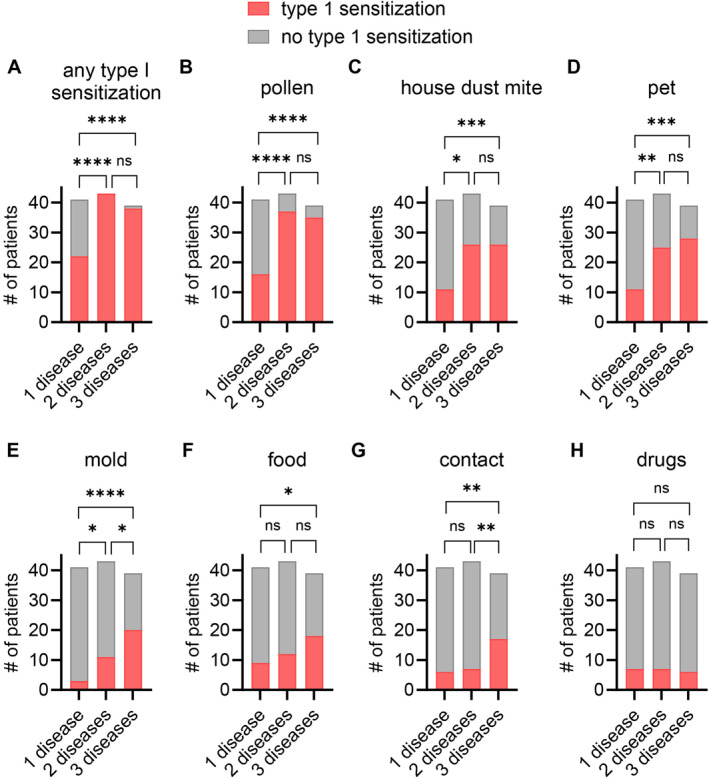
History of hypersensitivity reactions increases with high atopic disease burden. (A–H) Contingency plots indicate counts of patients with self‐reported allergic type I hypersensitivity (red colored) and without hypersensitivity (gray) against any allergen (A), pollen (B), house dust mite (C), pets (D), mold (E), food (F), contact (G) or drugs (H). *p‐*values were calculated using Fisher's exact test. *p < 0.05, **p < 0.01, ***p < 0.001, and ****p < 0.0001. ns, not significant.

Next, we wanted to see whether patients with multiple atopic diseases show differences in allergic sensitizations or intolerances. We assumed an increased abundance of allergic type I hypersensitivity reactions in patients with high atopic disease burden. Overall, reported allergic type I hypersensitivity reactions were significantly increased in individuals with more than one atopic disease (Figure 3A), with > 95% of patients with two or more diseases reporting allergic type I hypersensitivity against multiple allergens. We observed significantly higher numbers of individuals with allergic type I sensitizations with pollen, house dust mite and pets being the top three reported culprits of allergic type I hypersensitivity reactions (Figure 3B–D). Mold and food type I hypersensitivity as well as contact hypersensitivity was also significantly more frequently reported in patients with high atopic disease burden (Figure 3E–G). Surprisingly, drug hypersensitivity reactions did not increase with higher atopic disease burden (Figure 3H). These results confirm our hypothesis and suggest other factors to be involved in drug hypersensitivity than atopic disease burden alone.

### 3.4 IgE Increases With Atopic Disease Burden

**FIGURE 4:**
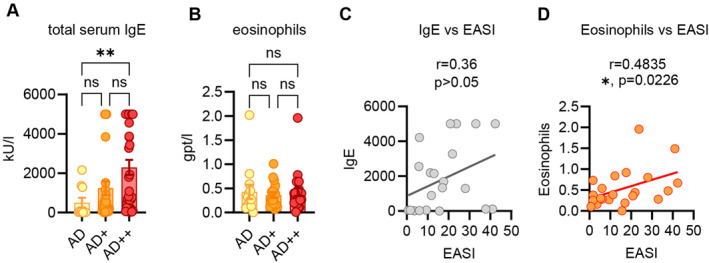
Enhanced serum IgE in atopic multimorbidity. (A, B) Comparison of total serum IgE or absolute eosinophil count (B) in AD patients with no (AD, *n* = 10), one (AD+, *n* = 26) or two (AD++, *n* = 30) classical atopic comorbidities. (C, D) Correlation plots of IgE (C) or eosinophil count (D) versus disease activity measured by Eczema Activity and Severity Index (EASI), Spearman coefficient. Mean + SEM. *p‐*values were calculated using ordinary one‐way analysis of variance (ANOVA) followed by Kruskal‐Wallis multiple comparison test. *p < 0.05, **p < 0.01, ns, not significant.

Given the increased allergic type I sensitization rates of patients with high atopic disease burden, we wanted to know whether this is reflected in laboratory results. AD is seen as the initial disease of the atopic march and represented the primary patient cohort of our University Hospital center. Therefore, we retrospectively assessed routine laboratory parameters that might be associated with atopic multimorbidity in AD patients. We categorized our cohort into AD only (*n* = 10), AD with one classical (AR or AA) atopic comorbidity (AD+, *n* = 26) and AD with two classical (AR and AA) comorbidities (AD++, *n* = 30). Interestingly, we saw a gradual increase of IgE levels from AD to AD++ with significantly elevated serum IgE in AD++ than AD only patients (Figure 4A), suggesting that high IgE is associated with atopic multimorbidity. We did not see differences in absolute eosinophil counts in AD++ patients compared to other groups. This is in line with previous studies where eosinophils have been rather associated with disease activity in AD [21] (Figure 4B). These findings were further corroborated by correlation analysis, showing no correlation between IgE and EASI (Figure 4C), but significant association of eosinophil count with EASI (Figure 4D).

### 3.5 T Cell Activation in AD Correlates Strongly With Disease Activity, But Not With Atopic Comorbidity

**FIGURE 5:**
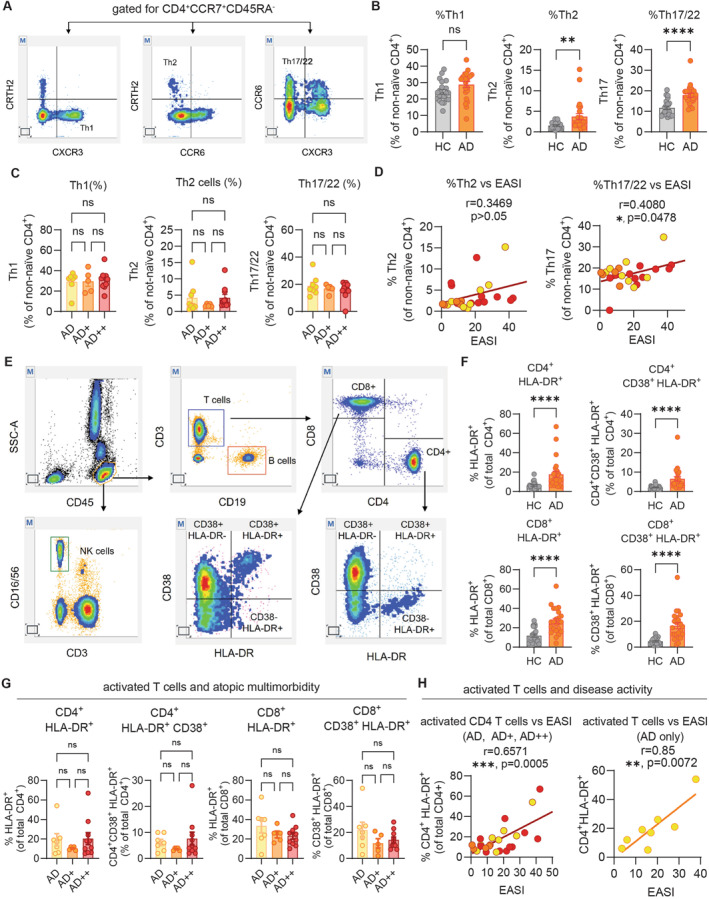
T cell activation in AD is independent of classical atopic comorbidity but correlates with disease activity. (A) Gating strategy for Th1, Th2 and Th17/22 cells. (B) Comparison of percentages of Th1, Th2 and Th17/22 in AD versus HC (*n* = 20). (C) Comparison of Th1, Th2 and Th17/22 cells in AD patients with no (AD, *n* = 7), one (AD+, *n* = 5) or two (AD++, *n* = 10) classic atopic comorbidities. (D) Correlation of disease activity (Eczema Activity and Severity Index, EASI) with percentage of Th2 or Th17/22 cells, Spearman coefficient. Color of dots correspond to AD, AD+ and AD++ as in C). (E) T cells were gated for CD4^+^CD8^‐^ and CD4^−^CD8^+^ and percentage of CD38 and HLA‐DR positivity among T cell subsets was assessed by flow cytometry. (F) Comparison of HLA‐DR^+^ and CD38^+^HLA‐DR^+^ T cells in AD (*n* = 22) versus HC (*n* = 20). (G) Comparison of activated T cells in AD, AD+ and AD++. (H) Correlation of EASI and activated T cells in AD, AD+ and AD++ (left, color of dots corresponds to AD, AD+ and AD++ as in G) or AD only (right). Pearson coefficient. Mean + SEM. *p‐*values were calculated using ordinary one‐way analysis of variance (ANOVA) followed by Šidák's multiple comparison test (C, G), Mann Whitney *U* test or Student's t‐test (B, F). *p < 0.05, **p < 0.01, ***p < 0.001, and ****p < 0.0001. ns, not significant.

Given that T cells are the main mediators of classical atopic disease pathogenesis, we then wanted to characterize the T cell immune landscape with flowcytometry‐based immunophenotyping prospectively in a subset of 22 patients with AD compared to 20 HC. First, we measured percentages of Th1, Th2 and Th17/22 cells in our cohort (Figure 5A). AD patients showed significantly higher percentages of Th2 and Th17/22 but not Th1 cells when compared to HC (Figure 5B). Importantly, Th1, Th2 and Th17/22 cells were similar in AD regarding comorbidities (Figure 5C). This indicates that despite a shared T cell‐driven immune response, we are unable to use the number of these cells to differentiate between low and high atopic disease burden. By contrast, the percentages of Th17/22 cells correlated significantly with EASI scores in AD (Figure 5D). Next, we wanted to assess the activation of peripheral T cells in patients with low and high atopic disease burden. We measured the percentage of CD38^+^ and HLA‐DR^+^ T cells, as these have been described to be increased in AD peripheral blood (Figure 5E) [6]. We confirmed highly significant and robust activation of both CD4^+^ and CD8^+^ T cells in AD compared to HC (Figure 5F). However, we did not see differences in patients with AD, AD + or AD++ (Figure 5G), suggesting that T cell activation is not associated with atopic disease burden per se. Importantly, activated T cells correlated well with EASI scores (Figure 5H, left), indicating that T cell activation in AD is rather reflective of disease severity than comorbidity. We also saw positive correlation of activated CD4^+^ T cells with patient‐reported ADCT (Supporting Information S1: Figure S1C). Total IgE did not correlate with activated T cells (Supporting Information S1: Figure S1D). To rule out that coexistence of AR or AA might skew these differences, we correlated T cell activation with EASI scores in individuals with AD only. Strikingly, we observed a strong and significant correlation despite relatively small patient numbers (Figure 5H, right), further corroborating our previous results.

Together, we show that individuals with AD and atopic multimorbidity exhibit higher rates of sleep disorder, type I sensitizations and higher total serum IgE values than individuals with AD only. By contrast, our data show that T cell activation peripheral blood is rather associated with individual disease activity in AD than atopic multimorbidity.

## 4 Discussion

Atopic multimorbidity defines the co‐occurrence of multiple atopic conditions within the same individual [2, 3]. The three classic atopic diseases (AA, AR, AD) as well as several others, that is CSU, food allergy or eosinophilic esophagitis, share features of type‐2 inflammation [1, 2, 22]. Our study highlights polysensitization, high IgE levels and psychological comorbidities in individuals with atopic multimorbidity.

We found differences between classical atopic diseases (AD, AR, AA) and CSU, with regards to age of onset and symptom duration. These differences might be explained by differences in pathogenesis: While all of these diseases share aspects of cellular drivers and Th2‐mediated inflammation with increases in cytokines related to type 2 immunity, the cell type‐specific signatures differ: Classical atopic diseases are primarily mediated by T cells, which secrete proinflammatory cytokines such as IL‐4, IL‐5, and IL‐13 that promote inflammation [4, 5, 6]. These cytokines also drive B cell‐mediated class switching to IgE, resulting in allergic sensitization and the hypersensitivity reactions that are characteristic of atopic disorders. In AD, type 2 cytokines promote downregulation of antimicrobial peptides and barrier proteins in keratinocytes as well as sensitization of neurons to pruritogens (e.g., IL‐31) in the skin [4]. Importantly, blocking type 2 cytokines through monoclonal antibodies targeting IL4Ra and IL‐13 was shown to be an efficient treatment strategy in AD [23, 24, 25]. Additionally, the anti‐IgE antibody omalizumab has significantly reduced AD severity in pediatric populations [26, 27], but has not been approved for AD due to lack of efficacy in the majority of the adult population. While pathogenesis of CSU is not fully understood, mast cell activation is recognized as a central component of disease pathogenesis, leading to histamine release [4]. Mast cell activation occurs via both IgE‐dependent (type I/autoallergic) and IgE‐independent (type IIb/autoimmune) mechanisms [28]. After mast cell degranulation, type 2 cytokines such as IL‐4 and IL‐13 contribute to B cell activation, IgE production and itch [4, 28]. Therapeutically, omalizumab is effective in a large subgroup of CSU patients, and more recently, the anti‐IL4Ra antibody dupilumab has also shown efficacy in CSU [29, 30]. Thus, although AD and CSU share overlapping targets and pathophysiological pathways—namely, type 2 cytokine signaling—differences in the specific cell types mediating these conditions may account for variation observed in our study.

Regarding non‐atopic comorbidities, we found high rates of patients with depression, anxiety and sleep disorders, the latter associated with high atopic disease burden. These results are in line with previous observations reporting different psychological comorbidities in AD that should be assessed when treating patients with atopic diseases [12, 13, 14].

Our study further highlights polysensitization in individuals with high atopic disease burden and higher total serum IgE levels in patients with concomitant AR, AD and AA. This is in line with reports of atopic multimorbidity in individuals with AR, showing higher IgE levels in patients with high atopic disease burden [31]. This indicates that IgE‐targeting medication may be of value to decrease atopic multimorbidity. Indeed, treatment with omalizumab in patients with asthma and comorbid food allergies is accompanied by reduction of reactions against accidental food exposure [32, 33]. By contrast, despite strong activation of T cells in these diseases, the levels of activated T cells did not differ between individuals with one compared to multiple atopic diseases. Based on our data, it seems to be rather individual disease activity that contributes to T cell activation [6, 34]. Interestingly, both CD4^+^ and CD8^+^ T cells were found to be activated in AD peripheral blood. We recently described enhanced STAT5 signaling in peripheral blood T cells in AD [34, 35], which might contribute to activation and was shown to promote an endurable effector‐like state in CD8^+^ T cells [36, 37]. The corresponding subset of Th2 cells in the CD8^+^ compartment that produces type 2 cytokines are Tc2 cells, which were shown to be increased in AD peripheral blood [5]. Therefore, it is intriguing to hypothesize that activated CD8^+^ T cells will undergo Tc2 differentiation.

Severity of AD has been reported to be associated with atopic comorbidities in a cohort of patients from the United States [38]. Associated comorbidities in AD include food allergy, anxiety, depression, autoimmune diseases and cardiovascular diseases [12, 39]. Importantly, severe AD showed stronger associations with these comorbidities than mild/moderate AD [38]. Our study showed higher T cell activation in more severe AD but not in atopic multimorbidity, reflected by coexistence of AA and AR. Unfortunately, we were underpowered to assess other comorbidities and their relationship to T cell activation (e.g., food allergy) but found strong association of T cell activation with disease severity, also strongly reflected in patients with AD only. Patients with atopic multimorbidity in our cohort did not show enhanced disease severity per se, but higher total IgE values, which was also reported in individuals with AR and other classic atopic comorbidities [31]. Our study used physician‐based assessment of disease activity reflected by EASI, which is independent of patient‐reported outcomes. We also saw associations with patient‐reported ADCT, corroborating our findings. Further studies with different study designs (e.g., longitudinal), including both physician‐ and patient‐reported outcomes and higher patient numbers are needed to elucidate these differences in T cell activation and disease severity‐based association with classic atopic and non‐atopic comorbidities. Furthermore, our results reflected T cells of peripheral blood in AD, but site‐ and disease‐specific patterns of T cell activation need further investigation. Overall, our study gives first insights into disease associations and laboratory findings of atopic comorbidity that need to be confirmed.

Limitations of our study include the rather small number of patients that have been investigated and low sample numbers for subset analysis. Our analysis was limited to assessing associations of classical atopic comorbidities while we were underpowered to test for other diseases. Furthermore, we did not assess temporary changes (e.g., childhood to adults) in our study. Multicentric longitudinal studies are needed to confirm our results, both of disease associations and laboratory findings.

## Author Contributions

**Ariane Bialas:** methodology, formal analysis, visualization, investigation, data curation, writing – original draft, software, validation. **Marie Rabe:** writing – review and editing, investigation, formal analysis, project administration. **Niklas Artz:** investigation, resources, writing – review and editing. **Andreas Boldt:** investigation, writing – review and editing, software. **Jan C. Simon:** writing – review and editing, investigation, supervision. **Regina Treudler:** writing – review and editing, supervision, resources, investigation. **Benjamin Klein:** conceptualization, methodology, writing – review and editing, funding acquisition, investigation, data curation, formal analysis, visualization, project administration, software, validation.

## Funding

This study was supported by the German Research Foundation (KL3612/2‐1) and from Hautnetz Leipzig/Westsachsen e.V. (942000‐245).

## Conflicts of Interest

B.K., A.r.B., M.R., and A.B. declare no conflicts of interest. N.A. reports personal fees from Sun Pharma. R.T. reports grants and personal fees from Sanofi‐Genzyme and Novartis, personal fees from ALK‐Abello, Almirall, CSL Behring, AbbVie, Pfizer, LeoPharma, Novartis, and Viatris, which are all independent of the submitted work. J.C.S. reports grants and personal fees from Sanofi‐Genzyme and Novartis, and personal fees from Lilly, Novartis, AbbVie, and LeoPharma.

## Supporting information


Supporting Information S1

## Data Availability

The data that support the findings of this study are available from the corresponding author upon reasonable request.
