# Supplementary material for: Atopic Multimorbidity in Adults With a Focus on Sensitization Patterns and T Cell Activation
Source: Clin Transl Allergy. 2025 Nov 28;15(12):e70129. doi: 10.1002/clt2.70129 (PMC12661119; doi:10.1002/clt2.70129)
Supplement: Supplementary file 1 — Supporting Information S1 [file CLT2-15-e70129-s001.docx]

**Supplemental Files**

**Supplemental Table 1. Summary of CSU patient characteristics (n=29).**

| Age | 44.6 (±16.3) |
| --- | --- |
| Female | 20/30 (66.7%) |
| Male | 10/30 (33.3%) |
| Positive ANA (≥1:80) | 14/30 (46,7%) |
| Positive TPO antibodies | 6/30 (20%) |
| Additional atopic disease (AD, AA, AR) | 17/30 (57%) |
| Total serum IgE | 307.1 kU/l (± 347.8) |

**Supplemental Figure 1.**


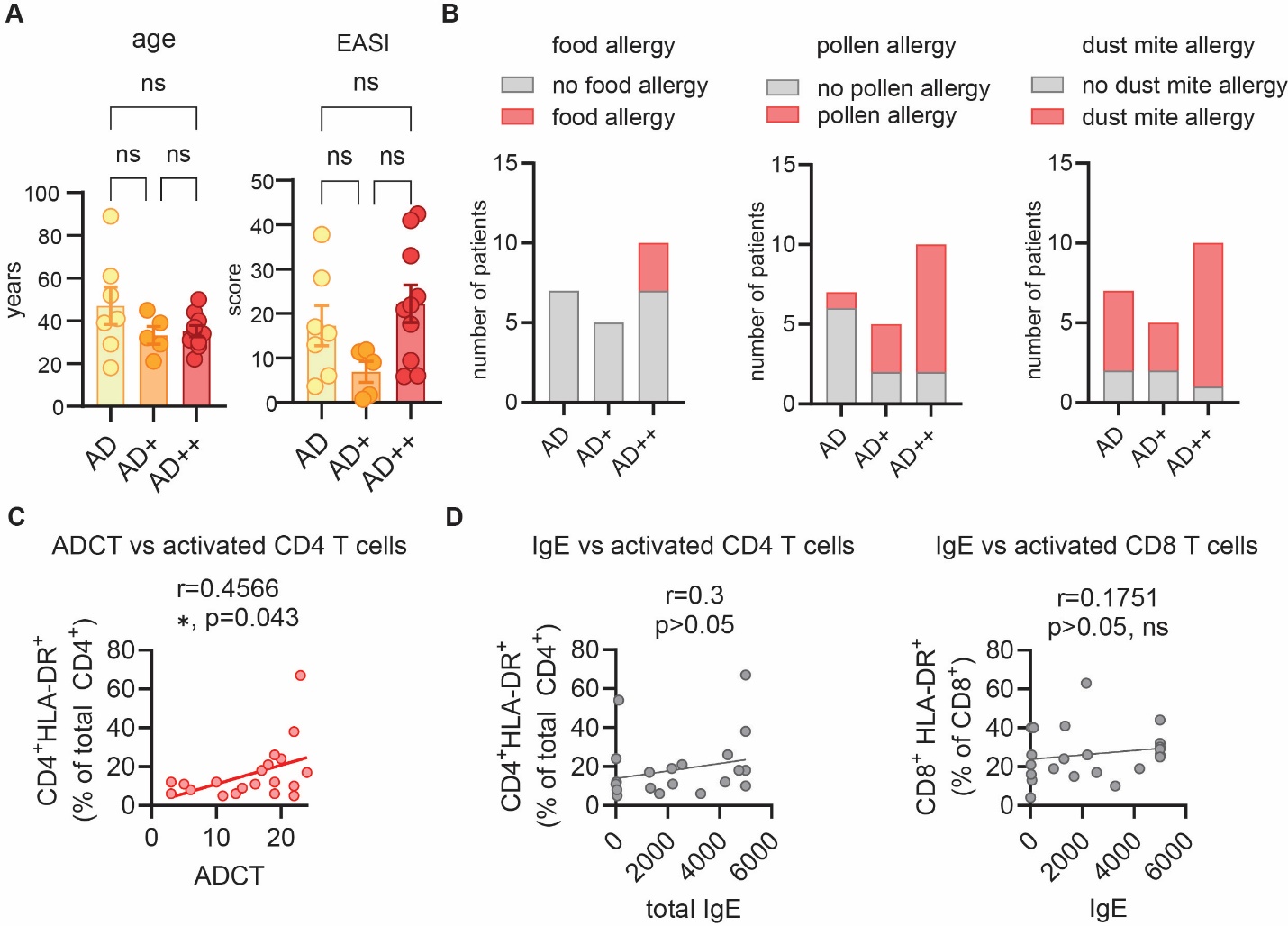


**Supplemental Figure 1.** **Disease activity and sensitizations in immunophenotyping cohort.**

**A**) Age and EASI scores in AD, AD+ and AD++ patients. **B**) Food, pollen and dust mite allergy in AD, AD+ and AD++. **C**) Correlation of Atopic Dermatitis Control Tool (ADCT) scores with percentages of peripheral activated CD4+ T cells. D) Correlation of total IgE with percentages of activated CD4^+^ T cells (left) or CD8^+^ T cells (right).
